# Development of an Efficient Virus Induced Gene Silencing Strategy in the Non-Model Wild Ginger-*Zingiber zerumbet* and Investigation of Associated Proteome Changes

**DOI:** 10.1371/journal.pone.0124518

**Published:** 2015-04-28

**Authors:** Chidambareswaren Mahadevan, Abdul Jaleel, Lokesh Deb, George Thomas, Manjula Sakuntala

**Affiliations:** 1 Division of Plant Molecular Biology, Rajiv Gandhi Centre for Biotechnology, Thycaud, Thiruvananthapuram, Kerala State, India-695014; 2 Proteomics Core Facility, Rajiv Gandhi Centre for Biotechnology, Thycaud, Thiruvananthapuram, Kerala State, India-695014; 3 Institute of Bioresources and Sustainable Development, Imphal, Manipur, India-795001; Nanjing Agricultural University, CHINA

## Abstract

*Zingiber zerumbet* (Zingiberaceae) is a wild, tropical medicinal herb that shows a high degree of resistance to diseases affecting cultivated ginger. *Barley stripe mosaic virus* (BSMV) silencing vectors containing an endogenous phytoene desaturase (*PDS*) gene fragment were agroinfiltrated into young leaves of *Z*. *zerumbet* under controlled growth conditions to effect virus-induced gene silencing (VIGS). Infiltrated leaves as well as newly emerged leaves and tillers showed visual signs of *PDS* silencing after 30 days. Replication and systemic movement of the viral vectors in silenced plants were confirmed by RT-PCR. Real-time quantitative PCR analysis verified significant down-regulation of *PDS* transcripts in the silenced tissues. Label-free proteomic analysis was conducted in leaves with established *PDS* transcript down regulation and buffer-infiltrated (mock) leaves. A total of 474 proteins were obtained, which were up-regulated, down-regulated or modulated *de novo* during VIGS. Most of these proteins were localized to the chloroplast, as revealed by UniprotKB analysis, and among the up-regulated proteins there were abiotic stress responsive, photosynthetic, metabolic and membrane proteins. Moreover, the demonstration of viral proteins together with host proteins proved successful viral infection. We report for the first time the establishment of a high-throughput gene functional analysis platform using BSMV-mediated VIGS in *Z*. *zerumbet*, as well as proteomic changes associated with VIGS.

## Introduction

*Zingiber zerumbet* Smith (Zingiberaceae) or ‘shampoo ginger’ is a rhizomatous perennial herb native to Southeast Asia. The rhizome of *Z*. *zerumbet* is rich in the triterpenoid zerumbone, which has demonstrated anti-inflammatory, anti-HIV [1] and anti-cancer [2] activities. *Z*. *zerumbet* expresses a high degree of resistance to the oomycete pathogen *Pythium* which causes the devastating soft-rot disease in cultivated ginger (*Zingiber officinale*) [3]. Ongoing efforts in our group have identified an ensemble of genes in *Z*. *zerumbet* that is regulated in response to *Pythium* inoculation [3,4]. These designated defense-gene candidates need to be functionally validated to assess their potential in future improvement programs for cultivated ginger.

Virus-induced gene silencing (VIGS) exploits an RNA-mediated antiviral defense mechanism. Though it normally targets viral genomes, the system can be manipulated to target host mRNAs by using virus vectors carrying inserts derived from the corresponding host genes [5]. Hence, the strategy has been used widely in plants for analysis of gene function and for high-throughput functional genomics [6,7]. Viral dsRNAs are identified by the plant’s defense machinery and are degraded into small interfering RNAs (siRNAs); in the case of the VIGS system, this leads to targeted down-regulation of the gene of interest [8]. The BSMV-mediated VIGS technique has been used for gene-silencing studies of monocots such as wheat [9] and barley [10]. It was also recently demonstrated that BSMV can infect two species within the Zingiberaceae, and BSMV–VIGS was applied to specifically down-regulate phytoene desaturase (*PDS*) in cultivated ginger [11], making it the first report on the application of BSMV-VIGS to *Zingiber* species in particular and non-cereal monocots as a whole. In the present study, we combined the BSMV-VIGS technique with *Agrobacterium*-mediated transient transformation of *Z*. *zerumbet* to down-regulate *PDS*, an endogenous marker gene widely used for VIGS studies in plants [12]. To our knowledge, this is the first report of such an attempt in *Z*. *zerumbet*. Our protocol could potentially accelerate the functional validation and characterization of candidate defense genes in *Z*. *zerumbet*.

*Z*. *zerumbet* being a highly pathogen-resistant ginger species, proteomic information obtained during its interactions with pathogens could help to elucidate possible relationships between the abundance of specific plant proteins and the plant’s response to pathogens. A comprehensive analysis at the protein level may also provide significant clues to the proteomic alterations induced by VIGS, which have not been reported so far. Thus, here we applied a mass spectroscopy (MS)-based label-free proteomics approach to gain insight into the plant global proteomic changes that define the virus–plant interaction associated with VIGS. Our study is the first to develop a VIGS strategy for down-regulation of a specific gene (encoding *PDS*) in *Z*. *zerumbet* and provides proteomic information in the context of VIGS. We also present a proof-of-concept experiment to show that proteomic changes associated with VIGS can be successfully identified by a simple label-free proteomic approach.

## Materials and Methods

### Plant material

**Fig 1:**
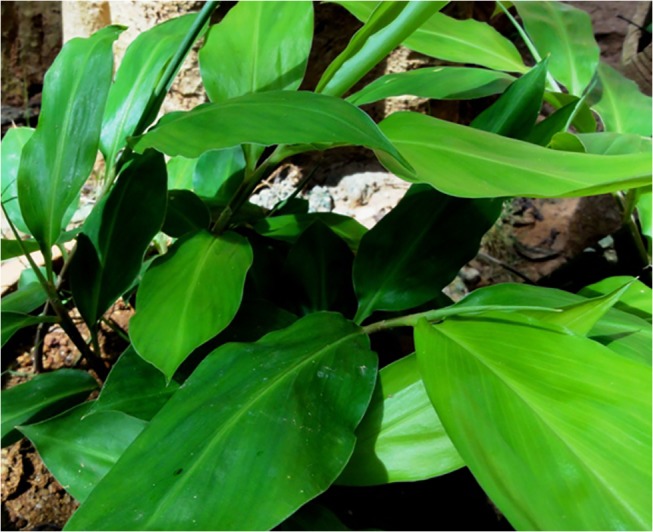
Young plant of *Zingiber zerumbet*.

*Z*. *zerumbet* accessions were maintained at Rajiv Gandhi Centre for Biotechnology, Thiruvananthapuram, Kerala, India. For VIGS experiments, young plantlets with two- or four-leaves (**Fig 1**) were selected and transferred to 20 cm × 14 cm×12.7 cm plastic pots filled with Soilrite mix (Keltech Energies Ltd, Bangalore), which is a mixture of 75% Irish peat moss and 25% horticultural grade expanded perlite. All plants were maintained in an environmental growth chamber (Model A1000 Conviron, Canada), and acclimatized for about one week at 24°C,70% RH and a photoperiod of 16h light and 8 h dark.

### Cloning of a phytoene desaturase (*PDS*) gene fragment from *Z*. *zerumbet*

Total RNA was isolated from 1g of leaf tissue of *Z*. *zerumbet* by the method of Salzman et al. [13]. DNase I (Ambion, Austin, TX) treatment was carried out to remove traces of genomic DNA. For cDNA synthesis, 2μg of total RNA was used along with oligo (dT) 15-mer primers (Promega, Madison, WI), 200 units of Moloney Murine Leukemia Virus (MMLV) reverse transcriptase (Promega, Madison, WI) and 40 units of ribonuclease inhibitor RNasin (Promega, Madison, WI). The *PDS* gene was amplified from *Z*. *zerumbet* using the primers ZPDS F3 and ZPDS R3 (**S1 Table**). PCR was carried out using Advantage 2 Polymerase mix (Clontech Laboratories, Inc., USA) with the following conditions: 94°C for 4 min; 35 cycles of 94°C for 30sec, 58°C for 30sec, 72°C for 1min; and 72°C for 4min and then held at 4°C. The PCR product was confirmed by gel electrophoresis prior to its cloning into plasmid pGEM T easy vector (Promega, Madison, WI). The nucleotide sequence of the cloned insert was then determined using the primer pairs T Vect F and T Vect R (**S2 Table**) in conjunction with Big Dye Terminator Cycle Sequencing (ABI).

### Phylogenetic analysis of *Z*. *zerumbet PDS*

Phylogenetic analysis of *Z*. *zerumbet PDS* gene homologs was performed by comparing EST sequences of *Z*. *officinale* (EY854183), *Lilium longiflorum* (AY 500373), *Crocus sativus* (AY 183118), *Zea mays* (L39266), *Oryza sativa* (AF 049356), *Triticum aestivum* (DQ 270236), *Hordeum vulgare* (AY 062039), *Hydrilla verticillata* (AY 639658) and *Lycopersicon esculentum* (S 36691). The phylogenetic tree was inferred using neighbor-joining methodology and tested by bootstrapping with 10,000 replications.

### Construction of *Z*. *zerumbet PDS*—containing BSMV-VIGS vector

The Agro/LIC BSMV-VIGS vectors used in this experiment were gifts from Dr. Dawei Li (State Key Laboratory of Agro-Biotechnology, China Agricultural University, Beijing, People’s Republic of China). Specific primers were synthesized with adaptor sequences as previously reported by Yuan et al. [14] for ligation-independent cloning (LIC). *Z*. *zerumbet*-*PDS* (*ZzPDS*) was PCR-amplified using the primer pair ZZPDSLIC F1 and ZZPDSLIC R1 (**S1 Table**). The empty vector pCaBSγb:00 was linearized with *Apa I* and used for LIC. Both the vector and the PCR product were eluted using a GFX PCR DNA and Gel Band Purification Kit (GE Healthcare).The linearized vector and the purified *ZzPDS* PCR product were treated with T4 DNA polymerase (New England Biolabs, MA, USA) in 1X reaction buffer containing 5mM dTTP and 5mM dATP separately at room temperature for 25min to generate complementary ends, and heated at 80°C to terminate the reaction. The pCa-γbLIC vector (40ng) was mixed with the *ZzPDS* LIC PCR product (~200-250ng), incubated at 65°C for 1-2min and slowly cooled at room temperature to anneal the complementary sequences. Later, 10μl aliquot was used for transformation into *Escherichia coli* DH5α (Invitrogen) competent cells, and transformants were identified by colony PCR using primer pairs, ZZPDS SP F1 and ZZPDS SP R1 respectively (**S1 Table**).

### Agroinfiltration of *Z*. *zerumbet* leaves

The BSMV-VIGS vectors pCaBS-α, pCaBS-β, pCaγb:00 (empty vector) and pCaγb:ZzPDS were transformed into *A*. *tumefaciens* strain GV3103 based on Weigel and Glazebrook [15]. For agroinfiltration, individual colonies of *Agrobacterium* were grown as 5ml cultures in LB (Luria and Bertani) medium (USB) containing Rifampicin (25 μg/ml) and kanamycin (100 μg/ml) (Sigma-Aldrich Co., St. Louis, MO), maintained overnight at 28°C on an orbital shaker (Kuhner, Switzerland). A 500-μl aliquot of each culture was used to inoculate 50ml LB containing the antibiotics described above and grown overnight at 28°C. The bacterial culture was pelleted at 3000g in a centrifuge (Jouan, MR23i, France) for 15 min at room temperature, resuspended in infiltration buffer containing 10 mM MgCl_2_, 10 mM 2-(*N*-morpholino) ethanesulfonic acid (MES) maintained at pH 5.2, additionally supplemented with 0.2 mM acetosyringone and incubated at room temperature for 2 h till ~0.6 OD_600_ was reached. Agroinfiltration was done by mixing equal amounts of the three bacterial cultures harboring pCaBS-α, pCaBS-β and pCa-γb:00 or pCaγb:*ZzPDS* and infiltrating the solution into 2–4 leaves of 3-month old *Z*. *zerumbet* plants using a 1-ml needleless syringe. The agroinfiltrated plants were maintained in a growth chamber (Conviron Model A1000) for 30days post-infiltration (dpi) at a temperature of 24°C, relative humidity of 70% and a photoperiod of16h light and 8h dark. Newly emerging leaves as well as leaves from newly arising tillers of the same rhizome were harvested at 15 and 30dpi and snap-frozen in liquid nitrogen and stored at -80°C for further analyses.

### RT-PCR for detection of viral movement

Total RNA was extracted from newly emerging leaves of mock, BSMV:00 empty vector and BSMV:*ZzPDS*—infiltrated test plants individually as described by Salzman et al. [13], and treated with RNase-free DNase I (Promega, USA). First-strand cDNA was synthesized using 2 μg of total RNA and a Superscript III cDNA Synthesis System (Invitrogen). RT–PCR for viral movement were carried out using BSMV γ-specific primers (BSMV GF1 and BSMV GR1; **S1 Table**). The PCRs were carried with the following conditions: 94°C for 4 min; 35 cycles of 94°C for 30sec, 55°C for 30sec, 72°C for 1min; 72°C for 4min and then held at 4°C. The products were separated on a 1.0% agarose gel and the images were scanned and analyzed using a Molecular Imager Gel Doc XR System (Bio-Rad, USA).

### Real-Time quantitative—PCR (RT-qPCR) analysis

RNA was isolated from control (empty vector) and *ZzPDS*-infiltrated leaves of *Z*. *zerumbet* as described previously at 15 and 30dpi. cDNA was prepared from 2μg RNA. For *ZzPDS* RT-qPCR analysis, the primers ZZPDS SP F3 and ZZPDS SP R5 (**S1 Table**) were designed using Primer Premier 6.0 software (Premier Biosoft International, Palo Alto, CA, USA). For determining the expression level of *ZzPDS*, the cDNA was diluted 10-fold and RT-qPCR was carried out by using Power SYBR Green PCR Master Mix (ABI) and SYBR Green I technology on a 7900 Real-Time PCR System (Applied Biosystems, Foster City, USA). A master mix was prepared and a 10-μl reaction mixture was set up in triplicate for each sample in a 96-well plate using thermal cycler conditions as recommended by the manufacturer. The housekeeping gene glyceraldehyde-3-phosphate dehydrogenase (*ZzGAPDH*), amplified using gene-specific primers (GAPDH2F and GAPDH2R; **S1 Table**), was used for normalization of the expression data. Data analysis was performed using RQ Manager SDS 1.2.1 software from ABI, and is presented as fold expression change in transcript accumulation. The values are the average obtained from two biological replicates, each performed in triplicate. The results from real-time qPCR were tested on GraphPad quickcalcs online software (www.graphpad.com/quickcalcs) for their statistical significance. Dissociation curve analysis was performed to rule out the presence of non-specific amplification and primer dimer formation.

### Protein extraction

Total protein was extracted as described earlier [16] with modifications. One gram of tissue from mock-infiltrated control leaves and BSMV *ZzPDS*-silenced leaves was ground to a fine powder using liquid nitrogen before protein extraction. Five to 10 mg of the lyophilized protein pellet was resolubilized in buffer composed of 7M urea, 2M thiourea, 40mM DTT and 1mM PMSF by vortexing for 30min at room temperature. The resolubilized protein was centrifuged at 14,000 g for 5min in a refrigerated centrifuge (Eppendorf, 5810R, Germany). The clear supernatant was transferred into a microcentrifuge tube. One hundred -200μl of the total protein was extracted by chloroform-methanol precipitation [17] and later solubilized in Rapigest SF (Waters) (0.1% (w/v) in 50 mM ammonium bicarbonate(Sigma) using a cycle of sonication, vortexing and heating to 95°C (5 min) to fully dissolve the pellet. Extracted proteins were quantified using a Bradford method [18]. A small portion (20 μg) from each sample was analyzed by SDS-PAGE to validate the protein estimation and ensure sample uniformity.

### Liquid chromatography–tandem mass spectrometry

Protein profiling and relative quantification (expression) analysis was done by Liquid Chromatography- tandem mass spectrometry (LC/MS/MS) by the following steps.

#### Preparation of tryptic peptides

Approximately 100 μg of protein from each sample was subjected to in-solution trypsin digestion to generate peptides. Briefly, protein disulfide bonds were reduced by treating the sample with 5μL of 100 mM DL-dithiothreitol in 50 mM ammonium bicarbonate for 30 min at 60°C and alkylated with 200mM iodoacetamide in 50 mM ammonium bicarbonate at room temperature for 30 min in the dark. Proteins were then digested by using trypsin (Sequence grade, Sigma) modified in 50 mM ammonium bicarbonate by incubating overnight at 37°C. The trypsin digestion reaction was stopped by adding 1 μL of 100% formic acid. The solutions containing digested peptides were centrifuged at 14000 rpm for 12 min, and the collected supernatant was stored at -20°C until LC/MS/MS analysis.

Before LC/MS/MS analysis of the peptide samples, appropriate peptide standards of various known proteins were spiked in different ratios among the samples for eventual protein expression analysis using a label-free method. For this, we used Mass PREP Digestion Standard (MPDS, Waters), a set of tryptic digested peptides from four proteins, namely, Bovine Serum Albumin (BSA), Rabbit Glycogen Phosphorylase b (GPB), Yeast Alcohol Dehydrogenase (ADH), and Yeast Enolase I (ENO). These MPDS (Waters) were available as Protein Expression Mixture 1(MPDS 1) and Protein Expression Mixture 2 (MPDS 2) having 1:2:8:0.5 fold abundance of peptides for ADH, ENO, BSA, and GPB, respectively, in MPDS 2. MPDS 1 was spiked in the control sample and MPDS 2 in the test sample.

#### Liquid chromatography

The peptide samples were analyzed by nanoLC–MS^E^ (MS at elevated energy) using a nanoAcquity UPLC system with 2D technology (Waters Corporation) coupled to a Quadrupole-Time of Flight (Q-TOF) mass spectrometer (SYNAPT-G2- HDMS^E^, Waters Corporation). Both systems were operated and controlled by MassLynx4.1 SCN781 software. In the 2D nanoLC, the peptides were separated by reverse phase (RP)/ reverse phase chromatography, one at high pH in the first dimension, followed by an orthogonal separation at low pH in the second dimension. Online dilution of the effluent was performed after the first dimension during trapping prior to the second dimension.

Briefly, 5μL of sample was injected in partial loop mode and was loaded into the first dimension column (RP column-1) with 20mM ammonium formate at pH 8 (aqueous mobile phase A) while unwanted solutes were washed to waste. The peptides were eluted from RP column-1 by the binary solvent manager-1 (BSM-1) into 3 different fractions sequentially based on %B (13.1%, 17.7% and 50%, respectively) and then trapped in the trap column. The organic mobile phase B was 100% Acetonitrile. To maximize sample recovery on the second dimension trap column from the organic-containing fractions, an aqueous flow of 0.1% formic acid in water at a flow rate of 20μL/min was delivered with the second dimension pump (BSM-2). Each fraction was resolved further in the second dimension RP column by the BSM-2. The second dimension separation was performed on a 75 μm × 100 mm BEH C_18_ 1.7 μm particle size column (Waters), with 1–40% B gradient, for 30 min at a 300 nL/min flow rate. After separation, the column was washed with 85% mobile phase B for 4 min and re-equilibrated with 1% mobile phase B for 14 min. The column temperature was maintained at 40°C.

#### Mass spectrometry

Mass spectrometric analysis of the eluting peptides from the LC was performed on a SYNAPT G2 High Definition Mass Spectrometer-HDMS^E^ (Waters). It is a hybrid, quadrupole, ion mobility, orthogonal acceleration, TOF mass spectrometer controlled by MassLynx4.1 SCN781 software.

The parameters used were the following: nanoESI capillary voltage, 3.3 kV; sample cone, 35 V; extraction cone, 4 V; transfer CE, 2 V. The TOF analyzer was calibrated with a solution of 500 fmole/μL of [Glu^1^]-fibrinopeptide B human and the lock mass acquisition was done every 30s by the same peptide delivered through the reference sprayer of the NanoLockSpray source at a flow rate of 500nL/min. This calibration set the analyzer to detect ions up to 2,000 *m/z*. Full mass spectra were acquired with a trap CE of 4 V. For mass to charge-selected ions (in resolving quad-mode), LM resolution was set to 4.7 and HM resolution to 15.0, with the quadrupole set to isolate the *m/z* of interest.

The experiment was run in Resolution mode, and the data format was continuum. Fragmentation was done in MS^E^ mode. Accurate mass data were collected in a data-independent mode of acquisition (MS^E^) by alternating between low (3 eV) and elevated (15−40 eV ramped) energy scan functions with no pre-selection of precursor ions by the quadrupole mass analyzer. The parameters were: Trap MS Collision Energy Low, (eV) 15; Trap MS Collision Energy High, (eV) 40; Transfer MS Collision Energy Low, (eV) 2; Transfer MS Collision Energy High, (eV) 2.0. For fragmentation in MS^E^ mode, the collision energy was linearly ramped in the Trap region of TriWave from 15 to 40 V across the duration of the high-energy scan. Generally, each spectrum was acquired for 0.9 sec.

#### Data analysis

The LC- MS^E^ data were analyzed by using ProteinLynx Global SERVER v2.5.3 (PLGS, Waters) for protein identification as well as for the label-free relative protein quantification. Raw data of all the fractions of *control* and *treated* were processed and then merged as single *control* file and *test* files. Data processing included *lock mass* correction post acquisition. Noise reduction thresholds for low-energy scan ion, high-energy scan ion and peptide intensity combined across charge states and isotopes were fixed at 150, 50 and 500 counts, respectively. The *Viridiplantae* reviewed database downloaded from *Uniprot* was used for the database search. During the database search, the protein *false positive rate* was set to 4%. A peptide was required to have at least two assigned fragments, and a protein was required to have at least one assigned peptide and five assigned fragments for identification. Oxidation of methionine was selected as a variable modification and cysteine carbamidomethylation was selected as a *fixed modification*. Trypsin was chosen as the enzyme used, with a specificity of 1 missed cleavage. Data sets were normalized using the ‘internal standard-normalization' function of Protein Lynx Global SERVER v2.5.3 and quantitative analyses were performed by comparing the normalized peak area/intensity of the identified peptides between the samples. ADH was chosen as the internal standard for normalization during expression analysis, and the top three intense peptides of ADH were used for that purpose. The differentially expressed protein data set was filtered by considering only those identified proteins that replicated in all three experimental replicates of each sample for each condition. Furthermore a 95% up- or down-regulation likelihood ([*P*>1]<0.05 or [*P*>1]>0.95) and a fold change higher than 50% (ratio of either <0.50 or >1.5) were considered to be indicative of significantly altered levels of expression.

## Results

### Cloning of *Z*. *zerumbet PDS* and VIGS vector construction

**Fig 2:**
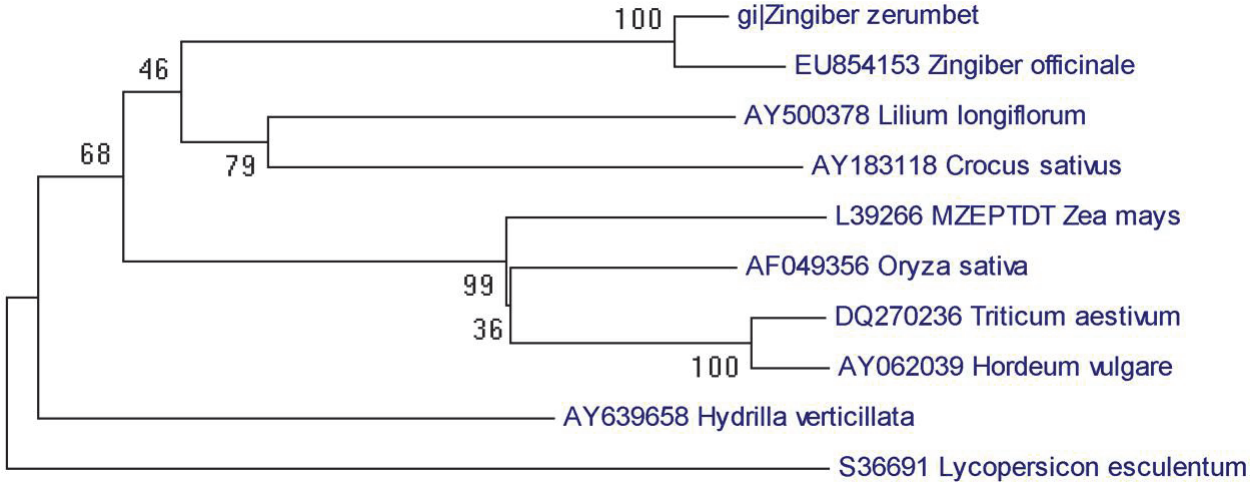
Phylogenetic analysis of *ZzPDS*. Phylogenetic analysis of *Z*. *zerumbet* phytoene desaturase (*PDS*) gene homologs was performed among EST sequences of *Z*. *officinale* (EY854183), *Lilium longiflorum* (AY 500373), *Crocus sativus* (AY 183118), *Zea mays* (L39266), *Oryza sativa* (AF 049356), *Triticum aestivum* (DQ 270236), *Hordeum vulgare* (AY 062039), *Hydrilla verticillata* (AY 639658), and *Lycopersicon esculentum* (S 36691). The phylogenetic tree was inferred using the Neighbor-joining method and tested by bootstrapping with 10000 replications. Bootstrap values >50% are shown at the nodes. The length of each branch represents the number of nucleotide substitutions per site.

*Z zerumbet PDS* gene-specific primers amplified a 380-bp fragment, and the amplified *ZzPDS* was cloned and sequenced. BLAST analysis revealed high sequence identity to several other reported *PDS* genes. *ZzPDS* showed 97% similarity to a partial coding sequence (cds) of *Z*. *officinale PDS* mRNA(EY854153.1)and 88% similarity to the complete cds of *Strelitzia reginae* (JX117875.1). Phylogenetic analysis revealed high genetic affinity between *ZzPDS* and *Z*. *officinale PDS* (**Fig 2**). Silencing vector pCaBSγb:*ZzPDS* was constructed using a ligation-independent cloning method as explained earlier. Gene-specific amplification of a 380-bp fragment confirmed the cloning of *ZzPDS* into the pCaγb:LIC vector. Subsequently, the binary vector was used to transform *Agrobacterium tumefaciens* GV3013, which was confirmed using colony PCR.

### Agroinfiltration and VIGS

**Fig 3:**
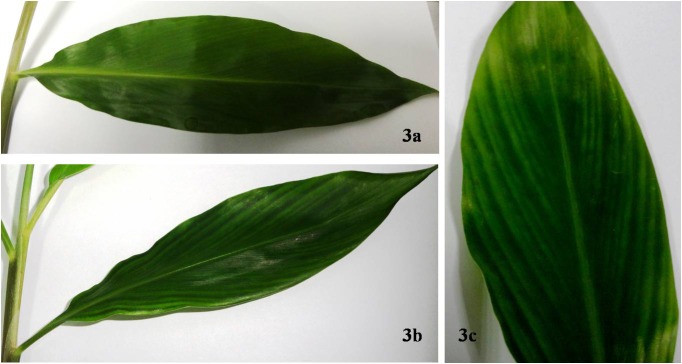
Agroinfiltration in *Z*. *zerumbet* leaves. **Fig 3a**. Control leaf of infiltrated *Z*. *zerumbet*. Agroinfiltration was carried out by mixing equal amounts of the three *Agrobacterium* (GV3103) cultures harboring pCaBS-α, pCaBS-β and pCa-γb: 00.The cultures were infiltrated into 2–4 leaves of 3-month *Z*. *zerumbet* plants using a 1-ml needleless syringe. The agroinfiltrated plants were maintained in a growth chamber for 30 dpi. Control leaf showed no visual signs of viral infection. **Fig 3b.** Leaves infiltrated with equal amounts of *Agrobacterium* cultures harboring pCaBS-α, pCaBS-β and pCaγb:*ZzPDS*. After 30dpi, yellow stripes were visible along the parallel veins, indicating sites of *PDS* down-regulation. **Fig 3c.** A closer view of a *PDS-*silenced leaf of *Z*. *zerumbet*.

**Fig 4:**
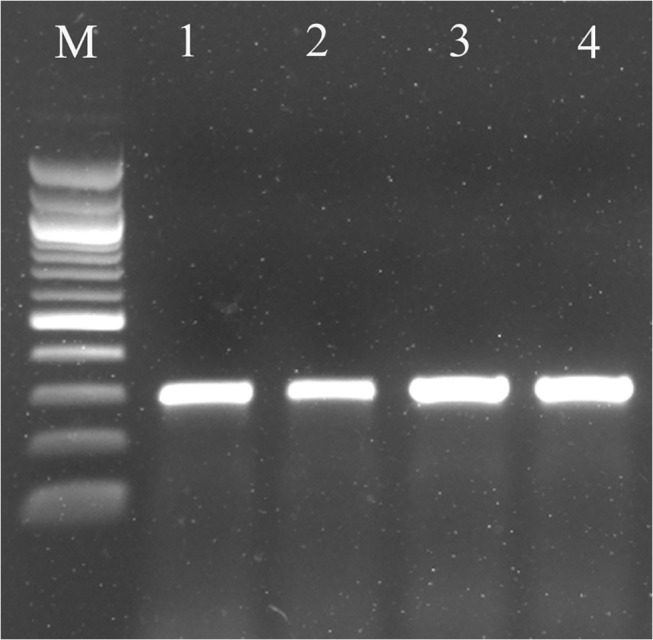
Detection of BSMV in silenced leaves of *Z*. *zerumbet* by RT-PCR. RNA was isolated from newly emerged leaves 30 days after infiltration with empty vector (control) and *ZzPDS*. Leaves were collected from the newly formed tillers as well. cDNA was prepared and RT-PCR was carried out using BSMV γ-specific primers. An amplicon of 309 bp representing BSMV γ transcript was obtained in all samples. M- 100bp ladder (NEB); Lane 1- Empty vector control; Lanes 2 & 3- Newly formed leaves of BSMV: PDS infiltrated plant 15dpi and 30dpi respectively; Lane-4- Leaf from new tiller 30dpi.

**Fig 5:**
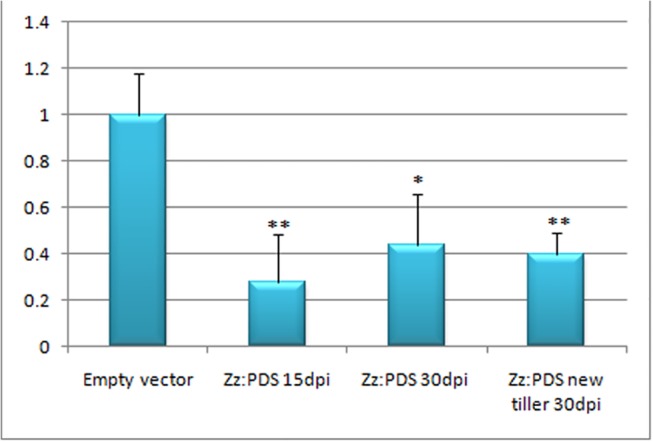
Real-time PCR analysis of PDS transcript accumulation in control and silenced leaves. Total RNA from control (empty vector) and *PDS*-silenced leaves of *Z*. *zerumbet* were isolated by the method of Salzmann et al. (1999) 15 and 30dpi. Quantitative real-time PCR was conducted using gene-specific primers of the *ZzPDS* gene to analyze its relative expression in leaf samples. *ZzGAPDH* was used as an internal control to normalize the samples. Values are means of two biological replicates performed in triplicate. *p < 0.05, **p < 0.01. X-axis- Leaf samples analyzed (from extreme left) 1- Empty vector-infiltrated (control); 2- Newly formed leaves of *ZzPDS*-infiltrated plant (15 dpi); 3- Newly formed leaf of *ZzPDS*-infiltrated plant (30dpi); 4- Leaf from new tiller developed from a BSMV:*ZzPDS*-infiltrated plant (30dpi). All the three silenced leaf samples showed significant downregulation of *PDS* transcripts and the values were found to be statistically significant. Y-axis- Relative expression levels of *ZzPDS*

Gene silencing in *Z*. *zerumbet* was carried out by agroinfiltration of pCaBSα + pCaBSβ + pCaγb:*ZzPDS*on 3–4 leaves of *Z*. *zerumbet* plants. The control plants were agroinfiltrated with empty vector pCaBSα + pCaBSβ + pCaγb:00 (BSMV: 00). Biological replicates of potted plants were infiltrated for each experiment and maintained in a controlled growth chamber for 30 days before being sampled. More than 90% of the leaves of BSMV: PDS-infiltrated plants showed yellow-stripes of photobleaching along the parallel venation (**Fig 3A–3C**). At15 and 30dpi, newly emerged leaves developed an evenly striated mosaic phenotype, which was evident in the new tillers also. BSMV infection was confirmed by RT–PCR using primers targeting a 309-bp fragment of BSMV γ RNA (**Fig 4**). These results were consistently observed in the all the separate experiments. RT–qPCR analyses showed a 70% and 55% reduction in the transcript levels of endogenous *ZzPDS* at 15 and 30 dpi, respectively, in newly emerged leaves of infiltrated plants and 60% reduction in the newly emerged tillers (**Fig 5**). The results from real-time qPCR were found to be statistically significant with a p-value <0.05.The presence of viral replicase (γ RNA) was confirmed by RT-PCR in both control and *PDS*-infiltrated plants.

### Proteomic profiling in BSMV-infiltrated leaves of *Z*. *zerumbet*

**Fig 6:**
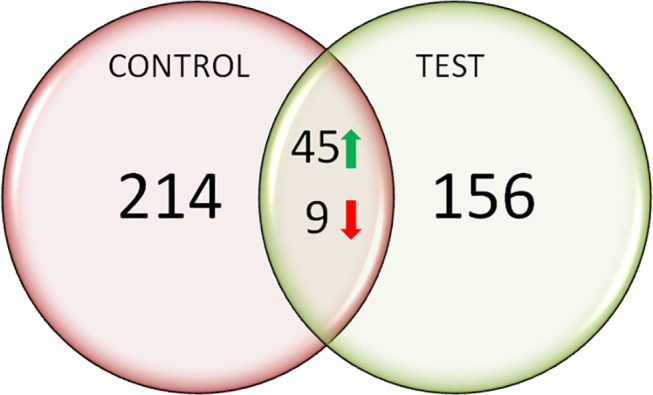
Venn diagram showing the number of proteins identified in the leaf proteome of *Z*. *zerumbet* in response to BSMV infection and *PDS* silencing. Total leaf protein was isolated by following the method of Isaacson et al. (2006) with some modifications, and proteomic analysis was carried out using LC/MS/MS. Among the474 proteins identified, 156 were expressed *de novo* in the silenced (tester) sample, 45 proteins were up-regulated and 9 down-regulated in response to VIGS. Proteomes of mock and *ZzPDS*-infiltrated leaves were profiled at 30 dpi.

The proteomes of mock and *ZzPDS*-infiltrated leaves were profiled at 30 dpi. There were only 20 reviewed proteins available in the UniprotKB database for Zingiberaceae. Therefore, we included all the proteins available in *Viridiplantae* for our relative quantitation and analysis. The database included all reviewed proteins of *Viridiplantae* (updated April 2013; 33735 reviewed proteins, 539829 sequences).In this study, LC-MS analyses yielded a total of 474 proteins that were up-regulated, down-regulated or expressed *de novo* during VIGS (**Fig 6**). The figure indicates distribution of the total proteins into control-specific (214), tester-specific (Zz*PDS* infiltrated) (156) and those differentially expressed in response to VIGS (45 upregulated and 9 downregulated).

### Functional characterization and subcellular localization

**Fig 7:**
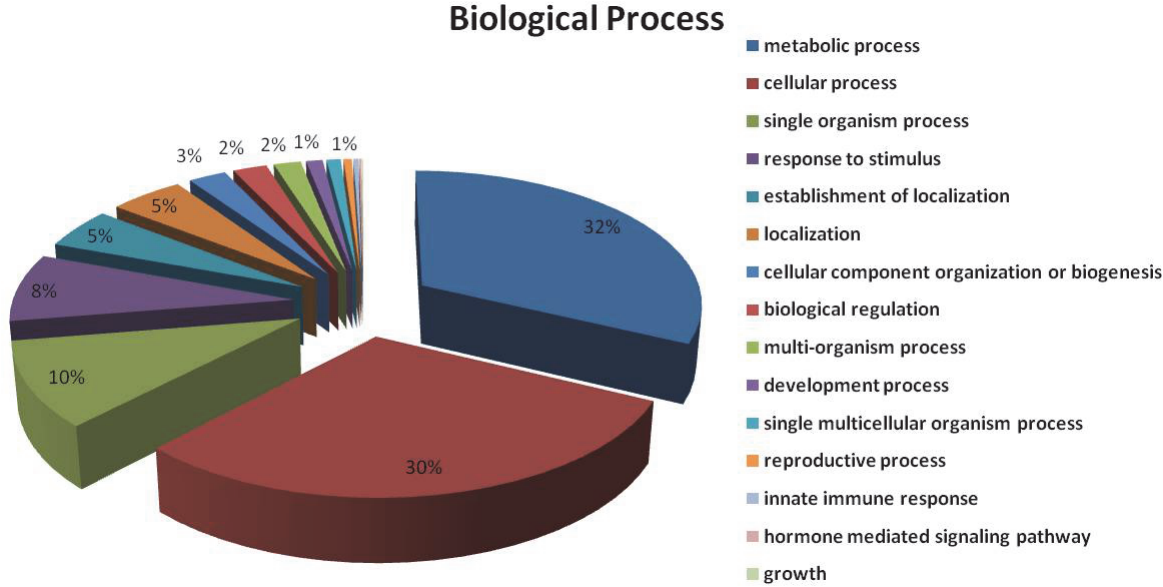
Distribution of the total proteins in the leaf proteome of *Z*. *zerumbet* identified by LC-MS/MS into biological processes according to annotations in UniProtKB. A major proportion of the identified proteins were predicted to be involved in metabolic (32%) or cellular processes (30%).

**Fig 8:**
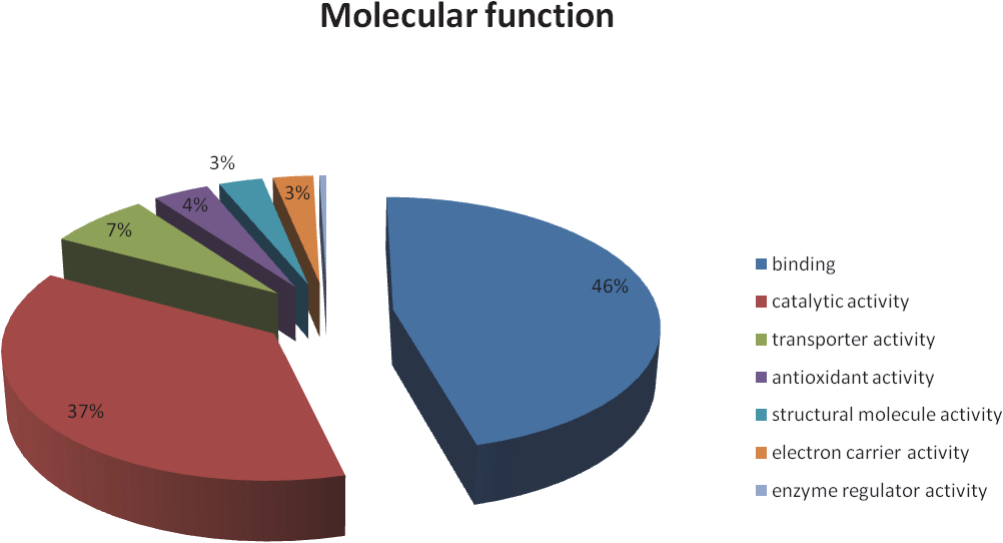
Categorization of the different leaf proteins of *Z*. *zerumbet* according to their predicted molecular functions based on annotations in UniProtKB. Nearly half of the total proteins identified (46%) were predicted to be associated with binding activity, closely followed by proteins showing catalytic functions (37%).

**Fig 9:**
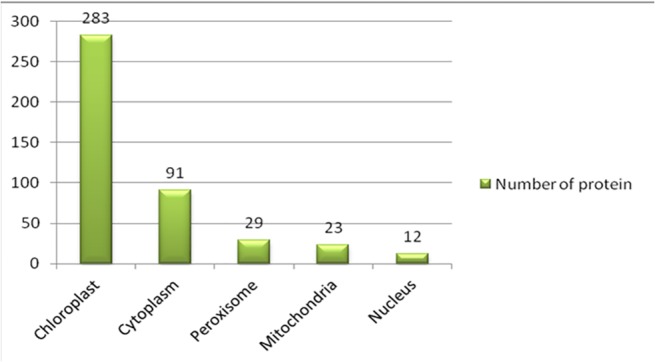
Organelle distributions of *Z*. *zerumbet* leaf proteins as predicted by UniProtKB. Data indicate that the majority of the proteins (283 proteins) were localized to the chloroplast compared to other organelles. The observation supports the hypothesis that BSMV infection directly regulates chloroplast-specific proteins and results in their upregulation.

The 474proteins identified were classified based on: (a) biological processes, (b) cellular component, and (c) molecular function according to annotations in UniProtKB. The subcellular localization of the total proteins was also identified using UniprotKB. The biological processes of the 474 proteins were classified into 15 functional groups, covering a wide range of functions. These include metabolic processes (32%), cellular processes (30%), single-organism processes (10%), response to stimuli (8%), establishment of localization (5%), cellular component organization (3%), biological regulation (2%) and developmental processes (1%) (**Fig 7**). The cellular component involved cell part (27%), organelle (25%), organelle part (15%), membrane (11%), macromolecular complex (11%), membrane part (8%), apoplast (2%), and intracellular organelle lumen (1%). The molecular functions include binding (46%), catalytic activity (37%), transporter activity (7%), antioxidant activity (4%), structural molecule activity (3%), electron carrier activity and enzyme regulator activity (3%) (**Fig 8**). Subcellular localization analysis predicted that a majority of the proteins identified were located in the chloroplast (64%), followed by cytoplasm (21%), peroxisomes (7%), mitochondrion (5%), and nucleus (3%) (**Fig 9**).

### Up-regulated VIGS-responsive proteins

Forty-five proteins were found to be up-regulated in response to BSMV-mediated *PDS* gene silencing in *Z*. *zerumbet* leaves (**S2 Table**). Most of the proteins identified have been previously reported in *Arabidopsis thaliana*, *Oryza sativa* subsp. *Japonica*, *Chlamydomonas reinhardtii*, *Hordeum vulgare*, *Nicotiana tabacum*, *Populus euphratica* and *Zea mays*. Most of the proteins are localized in the chloroplast, and functional classification of the up-regulated proteins suggests several important functions for these proteins, including glycolysis, tricarboxylic acid cycle, photorespiration, photosynthesis, embryo development, and defense response to abiotic and biotic stress. Ribulose 1, 5-bisphosphate [19], Nucleoside diphosphate kinase 1 [20], and Chlorophyll a-b binding proteins are important components of the PSII-associated light-harvesting complex. Fructose bis-phosphate aldolase (ALFC_PINST) [21] and Chloroplast phosphoglycerate kinase (PGKH_WHEAT and PGKH1_ARATH) [22,23] are chloroplastic proteins which have roles in photosynthesis and plastidic glycolysis. Ferredoxin NADP reductase (FENR_MESCR) [24] has an impact on photosynthetic performance by regulating electron transport in the chloroplast. A database created with Zingiberaceae and BSMV helped us identify 12 reviewed proteins of Zingiberaceae and 7 reviewed proteins of BSMV (**S3 Table**).

### Proteins specifically expressed *de novo* in response to VIGS

In principle, proteins which were expressed *de novo* in BSMV: PDS-infiltrated *Z*. *zerumbet* can be considered as VIGS-responsive proteins, and there were 156 such proteins (**S4 Table**). These proteins are localized mainly to the chloroplast, the mitochondrion, the peroxisome and the cytoplasm. Some had roles in electron transport (PSBA_ACOCL, PSBD_ADICA, CYF_AETGR, PSBD_ANGEV), and some were stress-responsive proteins like catalase (CATA4_SOYBN, CATA3_NICPL, CATA1_WHEAT, CATA2_WHEAT, CATA2_MAIZE), heat shock proteins (HSP70_SOYBN, HSFA9_ARATH, HSP7E_ARATH), and TH142_ SORBI. Other newly expressed proteins were ATP synthase, chlorophyll a-b binding protein, peptidyl-prolylcis-transisomerase, ferredoxin-NADP reductase, transcription factors, phosphoglucomutase, photosystenm Q(B) protein, oxygen-evolving enhancer proteins, rubilosebisphosphase carboxylase large and small subunits, ubiquitin-NEDD8-like protein, sedoheptulose-1,7-bisphosphate, serine-glyoxylate aminotransferase, tubulin beta, thiamine thiazole synthase, transketolase, triosephosphateisomerase, polyubiquitin8, mitochondrial outer membrane proteins, and plastocyanin proteins.

## Discussion

VIGS is a technology that exploits an RNA-mediated antiviral defense mechanism. VIGS has been used widely in plants for analysis of gene function and has been adapted for high-throughput functional genomics [5,7]. This strategy avoids production of knockout mutants or stable RNA interference (RNAi) and can also be performed on species that are difficult to transform [25]. Sequence fragments from ‘genes-of-interest’ are inserted into VIGS vectors used to ‘infect’ healthy plants and the corresponding host mRNAs are selectively degraded during the infection process to result in transient silencing of the targeted genes. BSMV-VIGS has been standardized for several cereals such as millet (*Setaria italica*), maize, and several members of the genus *Triticum* [26]. Recently the BSMV-VIGS technique was successfully utilized for down-regulation of phytoene desaturase (*PDS*) in *Z*. *officinale* [11], wherein foliar signs of photobleaching and stunted plant were reported as a consequence of *PDS* down-regulation. *PDS* silencing results in reduced levels of photo-protective carotenoids, leading to rapid destruction of chlorophyll by photo-oxidation which subsequently results in a photobleached leaf phenotype [27]. In the present study, photobleaching signs in *Z*. *zerumbet*, however, did not encompass the entire leaf area and appeared more confined to the narrow stripes parallel to the leaf veins. A similar phenotype was observed in *PDS*-silenced leaves of wheat [9]. This is different from the typical foliar sign of *PDS* silencing as is evident in *Nicotiana benthamiana* and other model dicot species, and possibly reflects the difference in leaf architecture between monocots and dicots and /or the spread of the virus within the leaf tissues of monocots and dicots.

An interesting observation in the present study was the sustained presence of viral transcripts and viral proteins in the infiltrated plants up to 30 days even in the absence of any visual signs of viral infection. This and previous observations, that most known host-resistance mechanisms appears to target viral replication or movement, and that ‘extreme resistance’ may be characterized by the absence of viral replication or occurrence of replication at essentially undetectable levels [28], prompted us to assume that *Z*. *zerumbet* could be partially resistant to BSMV. Yet another interesting finding is the systemic spread of the virus along with the ‘silencing signal’ to the leaves of newly formed tillers. Plant viruses typically move from the mesophyll via bundle sheaths, phloem parenchyma and companion cells into phloem sieve elements, where they are translocated and unloaded at a remote site where further infection will occur [29,30]. Plasmodesmata provide symplastic connectivity between the epidermal/mesophyll cells and cells within the vasculature, including the sieve elements [30]. Correspondingly, short-distance spreading of ‘silencing signals’ via plasmodesmata is limited to within 10–15 cells [31], whereas spreading of the silencing signal over a long distance is mediated through the phloem, usually from source to sink, following the direction of phloem flow [32]. This phenomenon is well demonstrated in the present work where the silencing phenotype was evident in the leaves of newly formed tillers. As *Z*. *zerumbet* is predominantly asexually propagated through rhizomes, the systemic spread of silencing signal to new tillers may prove advantageous in maintaining silenced clones for several cycles of experimentation.

There is a dearth of information on the antiviral defense proteome of monocotyledons, especially *Zingiber* species. Label-free quantitative proteomics-based experiments have recently been used for elucidating the proteomic changes associated with viral infection in *O*. *sativa* [33], *N*. *benthamiana*, [34] and *A*. *thaliana* [35]. In the present study, peptides were separated by two-dimensional RP chromatography at high pH in the first dimension and low pH in the second dimension prior to data-independent MS analysis [36]. This multidimensional LC separation not only increases the coverage by providing higher resolving power and better fractionation of the peptide mixture, but it also minimizes co-elution of different peptides that can happen during 1D LC separation. MS^E^-based data acquisition provides a near 100% duty cycle as precursors and product ion data for isotopes of all charge states are collected across the entire chromatographic peak width. The correlation of precursor ions to their corresponding MS/MS spectra is achieved by a retention time alignment algorithm. This method also allows one to collect sufficient data points in low collision mode to quantify peak ion intensities and, at the same time, obtain fragmentation data in high collision mode for protein identification [37]. We have used a more straightforward solution to high-throughput relative protein estimation, which is label-free MS-quantification. The label-free method uses ion signal intensities acquired by MS to assess the amount of peptides within the sample. The area of each ion can be calculated by integrating the extracted ion chromatograms, and the relative differences between two samples can then be assessed by comparing the calculated areas of two ions with the same mass [38,39]. A combination of all of the above advancements was used in this study to explore the proteomic changes associated with BSMV-VIGS infection in *Z*. *zerumbet*. In our experiments, the successful replication and systemic movement of BSMV was evidenced by the presence of alpha and gamma proteins (Q65715_BSMV) in the infiltrated leaf proteome. These proteins contain the helicase and RNA polymerase (RDRP) motifs and comprise the essential virus-encoded components of BSMV RNA replicase [40].

A plant cell infected with an RNA virus undergoes extensive membrane and organelle rearrangements and these modifications are often associated with viral RNA replication complex formation, viral RNA translation and cell-to-cell virus transport [29]. The prevalence of metabolic, cellular and membrane proteins in infiltrated *Z*. *zerumbet* leaves presumably reflects these phenomena. Sub-cellular organelle localization of the identified proteins revealed a preponderance of chloroplast-located proteins in the leaf proteome. Replication of BSMV is thought to be associated with vesicles in proplastids and chloroplasts [41,42]. It is postulated that recruitment of BSMV RNA to plastids could be a mechanism for spatial separation of translation and replication of positive single-stranded RNA viruses like BSMV [42]. In our study, a general up-regulation of chloroplast-localized proteins involved in photosynthesis and respiration was observed in response to viral infiltration. A similar up-regulation of chloroplast-specific protein synthesis genes was observed in sweet potato transcriptome profiling in response to viral infection [43]. Induction of host genes involved in protein synthesis is thought to be related to the virus's lack of translational machinery, which forces it to use the translational machinery of the host plant [43]. Another characteristic observation in the present study was the significant overexpression of photosynthesis-related proteins (RCA_CHLRE, RBS_MUSAC, Q1ZZ91_ZINOF, CB21_POPEU) in silenced leaf compared to the mock control. It was earlier reported that the pattern of gene expression characterizing a plant–virus interaction is specific to a particular host response (i.e. susceptible *vs*. resistant) to a pathogen and that, during infection, susceptible plants are characterized by significant down-regulation of photosynthetic genes [44]. Correspondingly, it is postulated, based on transcriptomic data, that resistant plants show significantly higher expression of photosynthetic genes in response to viral infection [44]. The up-regulation of transcript level may also result in increased protein expression, and could explain the protein-level overexpression of photosynthesis-related proteins in silenced leaves of *Z*. *zerumbet*, which is an inherently resistant species. Interestingly, the protein phosphoglycerate kinase (PGK) (PGKH_WHEAT, PGKH1_ARATH), which was found to be up-regulated in response to virus infiltration and VIGS, was previously shown to be required for efficient accumulation of Bamboo mosaic virus (BaMV), which is an RNA positive strand virus [45]. The interaction between chloroplast PGK and BaMV RNA was shown and it was suggested that the protein could assist viral RNA in targeting the chloroplast membrane [45].

The BSMV:*PDS*-infiltrated leaves were confirmed to be *PDS*-silenced prior to proteomic analysis and hence the proteome obtained could also reflect the effect of *PDS* down-regulation. *PDS* is an enzyme involved in the biosynthesis of carotenoids that provide protection to plants from photo-oxidative damage. Carotenoids are well known for their free radical scavenging activities. Photosynthetic organisms from anoxygenic photosynthetic bacteria through cyanobacteria, algae, and higher plants, as well as numerous non-photosynthetic bacteria and fungi, produce carotenoids [46]. In plants, inhibition of *PDS* renders the plant susceptible to damage from exposure to light and oxygen [47] and thus leads to higher expression of chloroplast-localized stress-responsive proteins. These phenomena probably explain the prevalence and over-expression of stress-responsive catalase, Heat Shock Proteins and ATP-binding proteins in the silenced leaf proteome of *Z*. *zerumbet*. This inference is partially supported by the observation of Romero et al. [48] in *PDS*-silenced tomato leaves, which exhibited a stress response correlated to the absence of anti-oxidative carotenoids. They also observed a pleiotropic effect of *PDS* silencing, the strong induction of glycoalkaloids and several phenolic compounds.

Overall, our results indicate that the proteomic data obtained in *Z*. *zerumbet* are a fair representation of the global proteomic changes associated with VIGS and plant–virus interactions in general. Our work could be developed further as a new approach for understanding the regulation of proteins during VIGS. To our knowledge, ours is the first study to report changes in the proteome during a VIGS experiment. Moreover, this is the first proteomics-based study to be applied to Zingiberaceae. Thus, this newly developed platform provides a method for identifying the proteins involved in defense, developmental processes, and secondary metabolite production in *Zingiber* species.

## Supporting Information

S1 Table Primers used in the present study. (DOCX)

S2 Table Proteins up-regulated in silenced leaves of *Z*. *zerumbet*. P-values between 0.95 and 1 represent a 95% likelihood of upregulation.
(XLSX)

S3 Table Proteins identified based on database entries for Zingiberaceae and Barley stripe mosaic virus (BSMV). The successful replication and systemic movement of BSMV is evidenced by the presence of alpha and gamma replicase proteins in the infiltrated leaf proteome (shaded portion).
(XLSX)

S4 Table List of proteins which are expressed *de novo* in BSMV: PDS-infiltrated *Z*. *zerumbet*. These 156 proteins can be considered as VIGS-responsive proteins.
(XLSX)

## References

[1] Dai JR, Cardellina J, Mahon J, Boyd M. Zerumbone, an HIV-inhibitory and cytotoxic sesquiterpene of Zingiber aromaticum and Z. zerumbet. Nat Prod Lett.1997;10: 115–118. 10.1080/10575639708043725

[2] Sobhan P, Seervi M, Deb L, Varghese S, Soman A, Joseph J, et al Calpain and reactive oxygen species targets Bax for mitochondrial permeabilisation and caspase activation in zerumbone induced apoptosis. PLoS ONE.2013;8 10.1371/journal.pone.0059350 PMC3621898 23593137

[3] Kavitha P, Thomas G. Defence transcriptome profiling of Zingiber zerumbet (L.) Smith by mRNA differential display. J Biosci.2008;33: 81–90. 10.1007/s12038-008-0002-2 18376073

[4] Nair R, Thomas G. Molecular characterization of ZzR1 resistance gene from Zingiber zerumbet with potential for imparting Pythium aphanidermatum resistance in ginger. Gene.2013;516: 58–65. 10.1016/j.gene.2012.12.011 23262347

[5] Lu R, Martin-Hernandez A, Peart J, Malcuit I, Baulcombe D. Virus-induced gene silencing in plants. Methods.2003;30: 296–303. 10.1016/S1046-2023(03)00037-9 12828943

[6] Fantini E, Falcone G, Frusciante S, Giliberto L, Giuliano G. Dissection of tomato lycopene biosynthesis through virus-induced gene silencing. Plant Physiol. 2013;163: 986–998. 10.1104/pp.113.224733 24014574 PMC3793073

[7] Senthil-Kumar M, Mysore K. New dimensions for VIGS in plant functional genomics. Trends Plant Sci.2011;16: 656–665. 10.1016/j.tplants.2011.08 21937256

[8] Benedito VA, Visser PB, Angenent GC, Krens FA. The potential of virus-induced gene silencing for speeding up functional characterization of plant genes. Genet Mol Res. 2004;3: 323–341. 15614725

[9] Scofield S, Huang L, Brandt A, Gill B. Development of a virus-induced gene-silencing system for hexaploid wheat and its use in functional analysis of the Lr21-mediated leaf rust resistance pathway. Plant Physiol. 2005;138: 2165–2173. 10.1104/pp.105.061861 16024691 PMC1183404

[10] Bruun-Rasmussen M, Madsen C, Jessing S, Albrechtsen M. Stability of barley stripe mosaic virus-induced gene silencing in barley. Mol Plant Microbe Interact. 2007;20: 1323–1331. 10.1094/MPMI-20-11-1323 17977144

[11] Renner T, Bragg J, Driscoll H, Cho J, Jackson A Specht CD. Virus-induced gene silencing in the culinary ginger (Zingiber officinale): An effective mechanism for down-regulating gene expression in tropical monocots. Mol Plant. 2009;2: 1084–1094. 10.1093/mp/ssp033 19825682

[12] Senthil-Kumar M, Hema R, Anand A, Kang L, Udayakumar M Mysore KS. A systematic study to determine the extent of gene silencing in Nicotiana benthamiana and other Solanaceae species when heterologous gene sequences are used for virus-induced gene silencing. New Phytol. 2007;176: 782–791. 10.1111/j.1469-8137.2007.02225.x 17997764

[13] Salzman R, Fujita T, Zhu-Salzman K, Hasegawa P, Bressan R. An improved RNA isolation method for plant tissues containing high levels of phenolic compounds or carbohydrates. Plant Mol Biol Rep. 1999;17: 11–17. 10.1023/A:1007520314478

[14] Yuan C, Li C, Yan L, Jackson A, Liu Z, Han C, et al A high throughput barley stripe mosaic virus vector for virus induced gene silencing in monocots and dicots. PLoS ONE. 2011;6 10.1371/journal.pone.0026468 PMC3198768 22031834

[15] Weigel D, Glazebrook J. Transformation of Agrobacterium using the freeze-thaw method. CSH protocols. 2006;7: 1031–1036. 10.1101/pdb.prot4665 22484682

[16] Isaacson T, Damasceno C, Saravanan R, He Y, Catala C, Saladie M, et al Sample extraction techniques for enhanced proteomic analysis of plant tissues. Nat Prot. 2006;1: 769–774. 10.1038/nprot.2006.102 17406306

[17] Wessel D, Flugge U. A method for the quantitative recovery of protein in dilute solution in the presence of detergents and lipids. AnalBiochem.1984;138: 141143 10.1016/0003-2697(84)90782-6 6731838

[18] Bradford MM. A rapid and sensitive method for the quantitation of microgram quantities of protein utilizing the principle of protein-dye binding. Anal Biochem.1976;72: 248–254. 942051 10.1016/0003-2697(76)90527-3

[19] Eichelmann H, Oja V, Rasulov B, Padu E, Bichele I, Pettai H, et al Development of leaf photosynthetic parameters in Betula pendula Roth leaves: Correlations with photosystem I density. Plant Biology. 2004;6: 307–318. 10.1055/s-2004-820874 15143439

[20] Spetea C, Lundin B. Evidence for nucleotide-dependent processes in the thylakoid lumen of plant chloroplasts—an update. FEBS Lett. 2012;586(18):2946–54. 10.1016/j.febslet.2012.07.005 22796491

[21] Mininno M, Brugière S, Pautre V, Gilgen A, Ma S, Ferro M, et al Characterization of chloroplastic Fructose 1,6-bisphosphate aldolases as lysine-methylated proteins in plants. J Biol Chem. 2012;287: 21034–21044. 10.1074/jbc.M112.359976 22547063 PMC3375527

[22] McMorrow, Eileen M, Bradbeer JW. Separation, purification, and comparative properties of chloroplast and cytoplasmic phosphoglycerate kinase from barley leaves. Plant Physiol. 1990;93 2: 374–383. 16667476 10.1104/pp.93.2.374 PMC1062521

[23] Cheng SF, Huang YP, Chen LH, Hsu YH, Tsai CH. Chloroplast phosphoglycerate kinase is involved in the targeting of Bamboo mosaic virus to chloroplasts in Nicotiana benthamiana plants. Plant Physiol. 2013;163: 1598–1608. 10.1104/pp.113.229666 24154620 PMC3846135

[24] Shin M. How is Ferredoxin-NADP reductase involved in the NADP photoreduction of chloroplasts? Photosynthesis Research. 2004;80: 307–313. 10.1023/B:PRES.0000030456.96329.f9 16328828

[25] Cakir C, Gillespie M, Scofield S. Rapid determination of gene function by virus-induced gene silencing in wheat and barley. Crop Sci. 2010;50: S–77S–84. 10.2135/cropsci2009.10.0567

[26] Lee WS, Hammond-Kosack K, Kanyuka K. Barley stripe mosaic virus-mediated tools for investigating gene function in cereal plants and their pathogens: virus-induced gene silencing, host-mediated gene silencing, and virus-mediated overexpression of heterologous protein. Plant Physiol. 2012;160: 582–590. 10.1104/pp.112.203489 22885938 PMC3461540

[27] Kumagai M, Donson J, della-Cioppa G, Harvey D, Hanley K, Grill LK. Cytoplasmic inhibition of carotenoid biosynthesis with virus-derived RNA. Proc Natl Acad Sci U S A. 1995;92: 1679–1683. 10.1073/pnas.92.5.1679 7878039 PMC42583

[28] Kang BC, Yeam I, Jahn MM. Genetics of plant virus resistance. Annu Rev Phytopathol. 2005;43: 581–621. 16078896 10.1146/annurev.phyto.43.011205.141140

[29] Carrington J, Kasschau K, Mahajan S, Schaad M. Cell-to-cell and long-distance transport of viruses in plants. Plant Cell. 1996;8: 1669–1681. 10.1105/tpc.8.10.1669 12239357 PMC161306

[30] Santa Cruz S. Perspective: Phloem transport of viruses and macromolecules–what goes in must come out. Trends Microbiol. 1999;7: 237–241. 10366860 10.1016/s0966-842x(99)01508-5

[31] Bai S, Kasai A, Yamada K, Li T, Harada T. A mobile signal transported over a long distance induces systemic transcriptional gene silencing in a grafted partner. J Exp Bot. 2011;62: 4561–4570. 10.1093/jxb/err163 21652532 PMC3170550

[32] Tournier B, Tabler M, Kalantidis K. Phloem flow strongly influences the systemic spread of silencing in GFP Nicotiana benthamiana plants. Plant J. 2006;47: 383–394. 16771840 10.1111/j.1365-313X.2006.02796.x

[33] Maxwell KL, Frappier L. Viral proteomics. Microbiol. Mol Biol Rev. 2007;71: 398–411. 17554050 10.1128/MMBR.00042-06 PMC1899879

[34] Laliberte JF, Sanfaçon H. Cellular remodeling during plant virus infection. Annu Rev Phytopathol. 2010;48: 69–91. 10.1146/annurev-phyto-073009-114239 20337516

[35] Niehl A, Zhang Z. J, Kuiper M, Peck S. C, Heinlein M. Label-free quantitative proteomic analysis of systemic responses to local wounding and virus infection in Arabidopsis thaliana. J Proteome Res. 2013;12: 2491–2503. 10.1021/pr3010698 23594257

[36] Gilar M, Olivova P, Daly A, Gebler J. Two-dimensional separation of peptides using RP-RP-HPLC system with different pH in first and second separation dimensions. J Sep Science. 2005;28: 1694–1703. 10.1002/jssc.200500116 16224963

[37] Levin Y, Hradetzky E, Bahn S. Quantification of proteins using data-independent analysis (MSE) in simple and complex samples: A systematic evaluation. Proteomics. 2011;11: 3273–3287. 10.1002/pmic.201000661 21751355

[38] Silva J, Denny R, Dorschel C, Gorenstein M, Kass I, Li GZ, et al Quantitative proteomic analysis by accurate mass retention time pairs. Anal Chem. 2005;77: 21872200 10.1021/ac048455k 15801753

[39] Wiener M, Sachs J, Deyanova E, Yates N. Differential mass spectrometry: A label-free LC−MS method for finding significant differences in complex peptide and protein mixtures. Anal Chem. 2004;76: 60856096 10.1021/ac0493875 15481957

[40] Petty I, French R, Jones R, Jackson A. Identification of Barley stripe mosaic virus genes involved in viral RNA replication and systemic movement. EMBO J.1990;9: 3453–3457. 2209552 10.1002/j.1460-2075.1990.tb07553.x PMC552093

[41] Lin NS, Langenberg WG. Distribution of Barley stripe mosaic virus protein in infected wheat root and shoot tips. J Gen Virol. 1984;65: 2217–2224.

[42] Torrance L, Cowan G, Gillespie T, Ziegler A, Lacomme C. Barley stripe mosaic virus-encoded proteins triple-gene block 2 and gammab localize to chloroplasts in virus-infected monocot and dicot plants, revealing hitherto-unknown roles in virus replication. J Gen Virol. 2006;87: 2403–2411. 10.1099/vir.0.81975-0 16847137

[43] McGregor CE, Miano DW, LaBonte DR, Hoy M, Clark CA, Rosa GJ. Differential gene expression of resistant and susceptible sweetpotato plants after infection with the causal agents of sweet potato virus disease. J Am Soc Hort Sci. 2009;134: 658–666.

[44] Havelda Z, Varallyay E, Valoczi A, Burgyan J. Plant virus infection-induced persistent host gene downregulation in systemically infected leaves. Plant J. 2008;55: 278–288. 10.1111/j.1365-313X.2008.03501.x 18397378

[45] Lin JW, Ding MP, Hsu YH, Tsai CH. Chloroplast phosphoglycerate kinase, a gluconeogenetic enzyme, is required for efficient accumulation of Bamboo mosaic virus. Nucleic Acids Res. 2007;35: 424–432. 10.1093/nar/gkl1061 17169994 PMC1802604

[46] Armstrong GA, Hearst JE. Carotenoids 2: Genetics and molecular biology of carotenoid pigment biosynthesis. FASEB J. 1996;10: 228–237. 8641556 10.1096/fasebj.10.2.8641556

[47] Glaser E, Soll J. Targeting signals and import machinery of plastids and plant mitochondria In: Daniell H, Chase C, editors. Molecular Biology and Biotechnology of Plant Organelles, Springer, Dordrecht, 2004 pp. 385–418

[48] Romero I, Tikunov Y, Bovy A. Virus-induced gene silencing in detached tomatoes and biochemical effects of phytoene desaturase gene silencing. J Plant Physiol. 2011;168: 1129–1135. 10.1016/j.jplph.2010.12.020 21377758

