# Supplementary material for: Development of an Efficient Virus Induced Gene Silencing Strategy in the Non-Model Wild Ginger-Zingiber zerumbet and Investigation of Associated Proteome Changes
Source: PLoS One. 2015 Apr 28;10(4):e0124518. doi: 10.1371/journal.pone.0124518 (PMC4412686; doi:10.1371/journal.pone.0124518)
Supplement: S1 Table — (DOCX) [file pone.0124518.s001.docx]

**S1 Table.** **1**: Primers used in this study

| **Primers** | **Primer sequence (5′–3′)** | **Usage** |
| --- | --- | --- |
| ZPDS F3 | CTTATGTTGARGCYCAAGATGG | Degenerate primers for Amplification of *Z. zerumbet* PDS gene fragments |
| ZPDS R3 | GTGTTCTTSAGTTTTCKRTCAAAC |  |
| ZZPDS SP F1 | AGCAGGGTGTGCCAGATCGAGT | Specific primers for amplification  of ZzPDS |
| ZZPDS SP R1 | CCCTTCCACTCGTTAGGCAAAAGG |  |
| ZZPDS SP F3 | GGAAAAGCATGGCTCGAAGATGGC | ZzPDS specific primers for  RT-qPCR analysis |
| ZZPDS SP R5 | GCAACAAATGCTTCACCGTTCCA |  |
| T Vect F | GGGTTTTCCCAGTCACGACGT | Primers for colony PCR of ZzPDS cloned on pGEMT Easy vectors |
| T Vect R | CGCCAAGCTATTTAGGTGACAC |  |
| BSMV AF | TGGCTAGCGATGAGATTGTCCGC | Primers for amplification of  BSMV α fragment |
| BSMV AR | TCTCCAGAAATCGAAAACAAGCCGCC |  |
| BSMV BF | TGGTGGGTCCACGTAGAAGCCT | Primers for amplification of  BSMV β fragment |
| BSMV BR | AGTTGTGGCTCTTTTCGCTCGGT |  |
| BSMV GF1 | AGCAGGTCCCGTCCTTACGCT | Primers for amplification of  BSMV γ fragment |
| BSMV GR1 | TGTCCCGGTAAAGACCACGTTCA |  |
| ZZPDSLIC F1 | AAGGAAGTTTAAAGCAGGGTGTGCCAGATCGAGT | Primers for amplification of ZzPDS gene fragment along with Ligation independent cloning(LIC) adaptors |
| ZZPDSLIC R1 | AACCACCACCACCGTCCCTTCCACTCGTTAGGCAAAAGG |  |
| GAPDH2F | ATGTGTCTGTTGTGGATCTCACTGT | Primers for *Z. zerumbet* GAPDH  gene as endogenous control for  RT-qPCR analysis |
| GAPDH2R | CCCTTGAGATTGCCCTCTGA |  |
